# Bullous pemphigoid diagnosis: the role of routine formalin-fixed paraffin-embedded skin tissue immunochemistry

**DOI:** 10.1038/s41598-022-14950-z

**Published:** 2022-06-22

**Authors:** Harim Oh, Chul Hwan Kim, Yoo Jin Lee

**Affiliations:** Department of Pathology, Korea University Anam Hospital, Korea University College of Medicine, Seoul, Korea

**Keywords:** Biomarkers, Diseases, Medical research

## Abstract

The gold standard for diagnosing bullous pemphigoid (BP) is the detection of linear deposition of IgG and/or C3 at the dermoepidermal junction using direct immunofluorescence (DIF). Because DIF has several disadvantages, primarily the requirement for frozen specimens, we assessed the diagnostic value of immunohistochemical (IHC) staining for BP detection. Eighty-eight patients with bullous lesions were included in this study. IHC staining for C3d, C4d, and IgG was performed on 88 samples, which included specimens from patients with DIF-confirmed BP (n = 43), clinicopathologically suspected BP with negative DIF results (n = 9), and other bullous diseases (n = 36). Diagnosis based on positive results for C3d, C4d, or IgG in IHC staining detected 86% of DIF-confirmed BP cases. The sensitivity of IHC staining for the detection of DIF-confirmed BP cases and clinicopathologically suspected BP cases was similar to that of DIF (80.8% vs. 84.3%), but the specificity was higher (83.3% vs. 75.0%). Five of the nine clinicopathologically suspected BP cases were diagnosed using IHC staining. Thus, IHC staining of routine biopsy material could be an alternative method for diagnosing BP. IHC staining has considerable diagnostic potential, especially in cases with a high suspicion of BP, but negative or suboptimal DIF results.

## Introduction

Bullous pemphigoid (BP) is the most common autoimmune subepidermal bullous disease involving the skin and mucosa and occurs predominantly in elderly patients^1^. BP usually presents as multiple tense bullae of varying sizes, pruritic urticarial plaques, vesicles, and crusted erosions. To diagnose BP in patients with compatible clinical manifestations, a skin biopsy is performed from the edge of a recent bulla or the perilesional area. The histological features of BP include subepidermal non-acantholytic blisters with conspicuous eosinophilic infiltration in the blister cavity and dermis. Although these histological features are important in identifying an intraepidermal or subepidermal bullous disease, it is impossible to obtain an accurate diagnosis based on histological characteristics alone^1–3^.

The pathogenesis of BP involves the production of autoantibodies against BP230 and BP180, which are transmembrane antigens associated with the hemidesmosomes of basal keratinocytes and the lamina lucida^1, 4^. After immunoglobulin binds to target antigens, the complement cascade is immediately activated, and an inflammatory reaction involving eosinophils and neutrophils occurs. This response produces matrix metalloproteinases or proteases, which weaken the dermoepidermal junction (DEJ)^4^.

To diagnose BP, an immunopathological examination to identify the immunoglobulins and complements involved is essential. In direct immunofluorescence (DIF) of perilesional skin biopsy, linear IgG and/or C3 deposits along the DEJ have been identified as the gold standard for BP diagnosis^1–3^. DIF has been reported to have a sensitivity of 82–90.5%^5–8^ and a specificity of 98%^8^. However, because of false-negative DIF results, Fudge et al.^4^ recommended repeat biopsy in clinically suspected BP cases with negative DIF findings. Additionally, DIF has the disadvantage of requiring frozen specimens, which means that additional skin biopsies must be performed to obtain frozen samples. Furthermore, the interpretation of the findings and handling of the samples were challenging.

Stable components of the complement cascade, such as C3d and C4d, can be detected using immunohistochemical (IHC) staining^9^, and their diagnostic value for some diseases has been well studied. The identification of linear C4d deposits in the peritubular capillaries of post-transplanted kidneys by IHC staining is essential for diagnosing antibody-mediated allograft rejection^10, 11^. The diagnostic potential of IHC analysis of C3d and C4d using formalin-fixed paraffin-embedded (FFPE) tissue specimens for autoimmune bullous skin disease has been suggested in previous studies^3, 9, 12, 13^. Although these studies showed different sensitivities, they suggested that IHC staining could be a substitute for DIF staining.

IgG detected by DIF is a valuable marker for diagnosing BP; however, the usefulness of IgG detected by IHC staining has not been examined. However, according to Zhang et al.^14^, IHC staining for total IgG in FFPE tissue specimens has no diagnostic value because of the high background staining. A study on IgG expression in the normal epidermis demonstrated that both the epidermis and dermis were stained by IgG^15^. Moreover, strong staining of the cytoplasm of cells in the stratum spinosum and stratum granulosum and weak staining in the stratum basale and stratum corneum were observed. However, the DEJ, which is vital for diagnosing BP, was not stained with IgG. Thus, we hypothesized that IgG-stained DEJ might have diagnostic value for BP.

IHC staining is widely used clinically for FFPE tissues and may be an alternative to DIF. If autoimmune skin diseases can be diagnosed using this method, numerous problems can be resolved. With IHC staining, BP diagnosis may be possible when frozen tissue is unavailable. Although several studies have attempted to assess the usefulness of IHC staining for detecting IgG, C3d, and C4d in FFPE skin specimens, its accuracy remains unclear. Hence, the aim of this study was to investigate the accuracy of IHC staining of FFPE skin specimens as an alternative to DIF for diagnosing BP, and to suggest that IHC staining has considerable diagnostic potential, especially in cases with a high suspicion of BP but negative or suboptimal DIF results.

## Materials and methods

### Patients and samples

A retrospective study was performed on 88 patients with bullous skin lesions between April 2011 and May 2019. In all the 88 cases, FFPE and frozen biopsy tissues obtained via punch biopsy were concomitantly subjected to DIF staining. Clinical data including sex, age, symptoms, clinical impressions, and treatment were obtained. The median age of the patients was 70 years (range, 1–98 years); among the 88 patients, 45 were men (51.1%) and 43 were women (48.9%). The lesions were located in the head and neck (10 cases, 11.4%), lower extremities (29 cases, 33.0%), trunk (28 cases, 31.8%), and upper extremities (21 cases, 23.8%).

Of the 88 cases, 43 were diagnosed with BP by DIF and pathological examination. Histologically, these lesions showed subepidermal bulla with eosinophils, and DIF revealed linear deposition of IgG or C3 along the DEJ. Although nine cases were pathologically and clinically suspected to have BP and were treated as such, DIF was negative (n = 8) or suboptimal because of the absence of the epidermis in the frozen biopsy specimens (n = 1). The remaining 36 patients were clinicopathologically diagnosed with other bullous diseases, including epidermolysis bullosa acquisita (n = 1), pemphigus foliaceus (n = 5), pemphigus vulgaris (n = 7), linear IgA bullous dermatosis (n = 2), bullous systemic lupus erythematosus (n = 3), Hailey-Hailey disease (n = 1), bullous lichen planus (n = 1), Stevens-Johnson syndrome/toxic epidermal necrolysis (n = 2), contact dermatitis (n = 1), cutaneous vasculitis (n = 3), dermatitis herpetiformis (n = 3), viral infection (n = 6), and nonspecific bullous disease (n = 1).

The study protocol was approved by the Institutional Review Board of the Korea University College of Medicine and conducted in accordance with the ethical principles of the Declaration of Helsinki (IRB approval no: 2019AN0305, approval date: July 17, 2019). As this was a retrospective, non-interventional study, informed consent was not required.

### Direct immunofluorescence

Cryocut 3-μm-thick sections of frozen tissue were immersed in acetone for 30 min, air-dried, and washed in Tris buffer. Antibodies against C3d, C4d, and IgG (pre-diluted 760-2686, 760-2687, and 760-2680, respectively; Roche, Mannheim, Germany) were used. After incubation for 50 min, the slides were rinsed with Tris buffer and a cover slip was placed using glycerin for fluorescence microscopy (Olympus AX70, Tokyo, Japan). Images were acquired using a camera attached to an epifluorescence microscope (Olympus, Tokyo, Japan). The deposition of IgG, C3d, and C4d was evaluated, and the continuous linear fluorescence deposition along the DEJ was detected as BP.

### Immunohistochemical staining

FFPE tissue blocks were sliced into 4-µm-thick sections. The sections were deparaffinized in xylene three times for 5 min each and rehydrated for 5 min in a graded series of alcohol concentrations (100%, 95%, 80%, and 70%). Antigen retrieval was performed for C3d, C4d, and IgG antibodies. Trilogy (Cell Marque, California, USA) was heated in a pressure cooker at 121 °C for 15 min. The container was then cooled for 20 min at approximately 20 °C. To reduce nonspecific background staining, slides were incubated in a hydrogen peroxide block (Cell Marque) for 10 min. Subsequently, the slides were washed three times in Tris-buffered saline (TBS, pH 7.6) for 5 min and incubated with a protein block (Cell Marque) at approximately 20 °C for 5 min. The antibodies used were anti-C3d (1:100, ab136916; Abcam, Cambridge, UK), anti-C4d (1:50, ab36075; Abcam), and anti-IgG (1:500, ab109489; Abcam). The slides were incubated with the antibodies for 40 min at approximately 20 °C and washed three times in TBS for 5 min each. Primary antibody amplifiers were applied for 10 min before incubation. Secondary antibodies contained a horseradish peroxidase substrate (GBI detection kit, USA), and were applied for approximately 30 min at approximately 20 °C. After washing with TBS, the samples were stained with a diaminobenzidine chromogenic substrate and counterstained with Mayer’s hematoxylin (ScyTek, USA). The positive controls for C3d and IgG were tonsil tissue, and the positive control for C4d was kidney tissue. Two pathologists reviewed the slides and only linear staining of the DEJ was considered a positive result.

### Statistical analysis

DIF is the gold standard for diagnosing BP; therefore, the diagnostic value of IHC staining was assessed using DIF as a reference. The sensitivity, specificity, positive predictive value (PPV), and negative predictive value (NPV) of the IHC staining were calculated. Additionally, considering the possibility of false-negative DIF results, we included cases that were clinicopathologically highly suspected of having BP to compare the diagnostic value of DIF and IHC staining. Subsequently, we calculated the diagnostic value of IHC staining and DIF for DIF-confirmed and highly suspected BP cases.

Moreover, we compared the results of IHC staining and DIF for detecting the same marker, and the concordance rate between the results from the different methods was assessed using the ĸ coefficient. SPSS 20.0 statistical software (SPSS Inc., Chicago, IL, USA) was used for statistical analyses.

## Results

### IHC staining results

**Figure 1:**
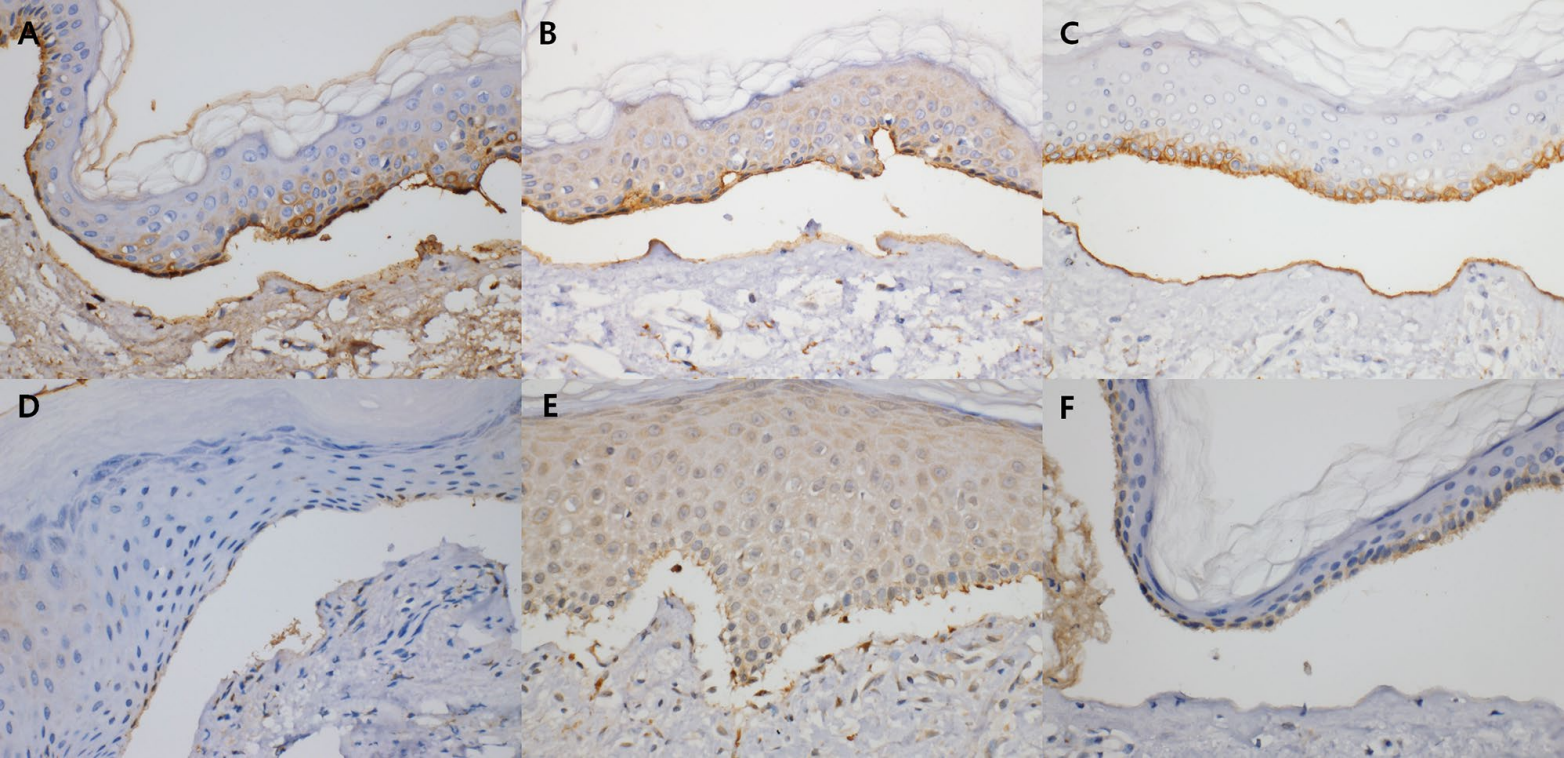
Immunohistochemical staining for IgG, C3d, and C4d using formalin-fixed paraffin-embedded tissue. Linear deposition of (**A**) IgG, (**B**) C3d, and (**C**) C4d along the dermo-epidermal junction. Negative staining for (**D**) IgG, (**E**) C3d, and (**F**) C4d (all panels: × 400).

IHC staining of specimens from 43 patients diagnosed with BP using DIF showed linear deposition of IgG in 28 cases, C3d in 21 cases, and C4d in 27 cases. Of the nine cases that could not be diagnosed as BP based on the DIF results, five showed linear deposition of IgG, two showed deposition of C3d, and two showed deposition of C4d (Fig. 1). Of the 36 cases in the other bullous disease group, six (linear IgA bullous dermatosis, n = 1; dermatitis herpetiformis, n = 1; viral infection, n = 2; bullous SLE, n = 1; and Stevens-Johnson syndrome, n = 1) showed linear deposition of IgG upon IHC staining. One case with a clinical impression of epidermolysis bullosa acquisita, but was negative by DIF, showed no linear deposition on IHC staining. Of three cases with clinically suspected bullous lupus erythematosus, two cases showed no linear deposition on IHC staining and one case showed linear IgG deposition on IHC staining. No case in the other bullous disease group showed linear deposition of C3d or C4d on IHC staining. These results are presented in Table 1.

**Table 1:** Immunohistochemical (IHC) staining results.

Group	DIF-confirmed		Highly suspicious		Other bullous disease	
	IF (n = 43)	IHC (n = 43)	IF (n = 8)	IHC (n = 9)	IF (n = 36)	IHC (n = 36)
IgG	31 (72.1%)	28 (65.1%)	0 (0%)	5 (55.6%)	2 (5.6%)	6 (16.7%)
C3d	39 (90.7%)	21 (48.8%)	0 (0%)	2 (22.2%)	9 (25%)	0 (0%)
C4d	10 (23.3%)	27 (62.8%)	0 (0%)	2 (22.2%)	0 (0%)	0 (0%)
IgG and/or C3d	43 (100%)	33 (76.7%)	0 (0%)	5 (55.6%)	9 (25%)	6 (16.7%)
C3d and/or C4d	39 (90.7%)	29 (67.4%)	0 (0%)	3 (33.3%)	9 (25%)	0 (0%)
IgG and/or C4d	32 (74.4%)	35 (81.4%)	0 (0%)	5 (55.6%)	2 (5.6%)	6 (16.7%)
IgG and/or C3d and/or C4d	43 (100%)	37 (86.0%)	0 (0%)	5 (55.6%)	9 (25%)	6 (16.7%)

### Diagnostic value of IHC staining

Based on the DIF results, parameters to determine the diagnostic value of IHC staining, including sensitivity, specificity, PPV, and NPV, were evaluated and are summarized in Table 2. The sensitivity, specificity, PPV, and NPV of IHC staining were 65.1%, 75.6%, 71.8%, and 69.4%, respectively, for IgG, 48.8%, 95.6%, 91.3%, and 66.2% for C3d, and 62.8%, 95.6%, 93.1%, and 72.9%, respectively, for C4d. The diagnostic parameter values differed when more than one marker was included in the diagnostic criteria. The highest sensitivity (86.0%) was noted when linear deposition was confirmed for C3d, C4d, or IgG, although the specificity (75.6%) was low. The specificity of IHC staining is low, particularly for IgG detection. However, when the diagnostic criteria included only C3d and/or C4d, IHC staining exhibited a lower sensitivity (67.4%) and higher specificity (93.3%).

**Table 2:** Diagnostic value of IHC staining based on DIF-confirmed BP cases.

	Sensitivity (%)	Specificity (%)	PPV (%)	NPV (%)
IgG	65.1	75.6	71.8	69.4
C3d	48.8	95.6	91.3	66.2
C4d	62.8	95.6	93.1	72.9
IgG and/or C3d	76.7	75.6	75.0	77.3
C3d and/or C4d	67.4	93.3	90.6	75.0
IgG and/or C4d	81.4	75.6	76.1	81.0
IgG and/or C3d and/or C4d	86.0	75.6	77.1	85.0

*BP* bullous pemphigoid, *IHC* immunohistochemical, *DIF* direct immunofluorescence, *PPV* positive predictive value, *NPV* negative predictive value.

### Comparison of the diagnostic value of DIF and IHC staining

Although DIF is the gold standard, false-negative DIF results have been observed. Thus, we included cases of clinicopathologically suspected BP as true BP cases to compare the diagnostic value of DIF and IHC staining. The results are summarized in Table 3.

**Table 3:** Comparison of the diagnostic value of IHC staining and DIF between the DIF-confirmed and highly suspicious groups.

	Sensitivity		Specificity		PPV		NPV	
	DIF (%)	IHC (%)	DIF (%)	IHC (%)	DIF (%)	IHC (%)	DIF (%)	IHC (%)
IgG	60.8	63.5	94.4	83.3	93.9	84.6	63.0	61.2
C3(d)	76.5	44.2	75.0	100	81.3	100	69.2	55.4
C4(d)	19.6	55.8	100	100	100	100	46.8	61.0
IgG and/or C3(d)	84.3	73.1	75.0	83.3	82.7	86.4	77.1	68.2
C3(d) and/or C4(d)	76.5	61.5	75.0	100	81.3	100	69.2	64.3
IgG and/or C4(d)	62.7	76.9	94.4	83.3	94.1	87.0	64.2	71.4
IgG and/or C3(d) and/or C4(d)	84.3	80.8	75.0	83.3	82.7	87.5	77.1	75.0

*BP* bullous pemphigoid, *IHC* immunohistochemical, *DIF* direct immunofluorescence, *PPV* positive predictive value, *NPV* negative predictive value.

Compared with DIF, IHC staining showed higher sensitivity (63.5% vs. 60.8%) but lower specificity (83.3% vs. 94.4%) for IgG, lower sensitivity (44.2% vs. 76.5%) but higher specificity (100.0% vs. 75.0%) for C3d, and higher sensitivity (55.8% vs. 19.6%) for C4d. IHC staining and DIF showed the same specificity (100.0%) for C4d. The highest diagnostic value of IHC staining was observed when the diagnostic criteria were IgG, C3d, and C4d. The sensitivity was similar to that of DIF (80.8% vs. 84.3%), while the specificity (83.3% vs. 75.0%) and PPV (87.5% vs. 82.7%) were higher for IHC staining. The NPV (75.0% vs. 77.1%) of IHC staining was lower than that of DIF staining.

Furthermore, of the nine cases of clinicopathologically suspected BP that were not diagnosed as BP owing to negative DIF results, five were diagnosed as BP using IHC staining. One of the five cases showed suboptimal DIF results because of the absence of epidermal tissue.

### Diagnostic value of two or more markers identified by IHC staining for BP

When the diagnostic criteria included the linear deposition of two or more markers detected by IHC staining, that is, C3d and IgG, C4d and IgG, C3d and C4d, or C3d, C4d, and IgG, 26 cases were diagnosed with BP (23 out of 43 confirmed BP cases, 3 out of 9 suspected BP cases, and 0 out of 36 cases of other bullous diseases). These diagnostic criteria exhibited high specificity (100%), but low sensitivity (50%). PPV and NPV were 100% and 58.1%, respectively.

### Diagnostic value of the combination of DIF and IHC staining

Because both DIF and IHC staining showed high sensitivity, we assessed the combination of these two detection methods and determined their diagnostic value. The combination showed a higher sensitivity (92.3%) but lower specificity (58.3%) than DIF or IHC staining alone. PPV and NPV were 76.2% and 84.0%, respectively.

### Comparison of DIF and IHC staining

Although DIF and IHC staining were performed on different specimens, we compared the expression of the evaluated markers as detected by DIF and IHC staining in 87 cases with available DIF results (Tables S1–S3). One patient was excluded because of suboptimal DIF results. Of the 33 IgG-positive cases detected via DIF, 20 (60.6%) revealed linear IgG deposition upon IHC staining. Of the 54 DIF-negative cases, 36 (66.7%) also exhibited negative IHC staining results. Of the 48 C3d-positive cases detected by DIF, 20 (41.7%) exhibited linear deposition of C3d by IHC staining, and 37 of 39 DIF-negative cases (94.9%) were also negative for C3d after IHC staining. Moreover, of the 10 C4d-positive cases detected by DIF, seven (70.0%) revealed linear deposition of C4d upon IHC staining, and 55 of 77 DIF-negative cases (71.4%) were negative for C4d upon IHC staining.

Discrepancies were observed between the DIF and IHC staining results. In 18 IgG-, two C3d-, and 22 C4d-positive cases, deposition was observed by IHC staining but not by DIF; 8 out of 18 IgG-, 1 out of 2 C3d-, and 20 out of 22 C4d-positive cases were diagnosed as BP using DIF, whereas 4 out of 18 IgG-, 1 out of 2 C3d-, and 2 out of 22 C4d-positive cases were considered clinically suspected cases. Thus, 12 of 18 (66.7%) IgG-, 2 of 2 (100%) C3d-, and all 22 (100%) C4d-positive cases were treated as BP cases. Of the 18 IgG-positive cases (33.3%), one was diagnosed with linear IgA bullous dermatosis, one was diagnosed with dermatitis herpetiformis, two were diagnosed with viral infections, one was diagnosed with bullous SLE, and one was diagnosed with Stevens-Johnson syndrome.

We also assessed the concordance rate between the methods using the ĸ coefficient. All the results showed a fair concordance rate, and the ĸ values for IgG, C3d, and C4d were 0.265 (*p* = 0.013), 0.344 (*p* ≤ 0.001), and 0.227 (*p* = 0.009), respectively.

## Discussion

Detecting linear deposition of autoantibodies and/or complements along the DEJ using DIF is essential for diagnosing BP. However, the need for frozen specimens in DIF is a disadvantage. In a routine examination, hematoxylin and eosin-stained slides of FFPE tissue were examined to identify histological features, and clinicians performed additional skin biopsies to obtain frozen samples for DIF testing. Additionally, given the nature of frozen specimens, obtaining samples suitable for DIF testing may be difficult, and although rare, false-negative DIF results are possible, which could lead to an inaccurate diagnosis. In this study, we hypothesized that IHC staining of complement components and IgG using FFPE tissue could be used to diagnose BP, thereby enabling immunopathological confirmation of the bullous process without the disadvantages of DIF.

We determined that 86% of BP cases diagnosed by DIF could also be diagnosed via the detection of C3d, C4d, or IgG deposition by IHC staining as the diagnostic criterion. Considering the possibility of false-negative DIF results, we compared the DIF and IHC staining findings, including those of clinicopathologically suspected BP cases with negative DIF results. Using the deposition of IgG, C3d, and C4d as diagnostic criteria, we found that IHC staining had high sensitivity (although it was slightly lower than that of DIF) and higher specificity than DIF. Thus, IHC staining may be a useful method for diagnosing BP, although DIF remains the gold standard.

When C3d deposition was used as the sole diagnostic criterion for IHC staining, the sensitivity was extremely low (44.2–48.8%); however, the specificity was high (95.6100%). Several studies on C3d IHC staining of FFPE skin biopsy specimens for BP diagnosis have reported high sensitivity (97–100%)^9, 12^. However, Glauser et al.^13^ demonstrated that sensitivity was only as high as 37%. Our study had the second largest number of cases after the study by Glauser et al.^13^, which reported a low sensitivity. Similarly, our results showed that IHC staining for C3d detection has low sensitivity for diagnosing BP.

C4d is an essential diagnostic marker for antibody-mediated kidney rejection, and the presence of C4d can be detected by either DIF or IHC staining^10, 11^. In BP cases, the sensitivity for C4d by IHC staining has been reported to be 24–90%^3, 9, 16^. Our study, which included the second largest number of BP cases reported to date, showed high sensitivity (55.8–62.8%) and high specificity (95.6–100%) for C4d detection. Thus, C4d could be a useful marker for BP based on IHC staining.

IgG detection by IHC staining of FFPE tissues for the diagnosis of BP has not been performed previously. Zhang et al.^14^ reported that IHC staining for IgG has no diagnostic value because of its high background staining. However, we hypothesized that detecting strong deposition of IgG along the DEJ by IHC could aid in diagnosing BP, regardless of background staining. We evaluated linear deposition only along the DEJ and found that IHC staining for IgG had a slightly higher sensitivity (63.5–65.1%) and slightly lower specificity (75.6–83.3%) than DIF. Thus, we suggest that the linear deposition of IgG, even in FFPE tissues, could be another diagnostic marker for BP when IHC staining is used.

Furthermore, five out of nine (55.6%) cases with negative DIF results were diagnosed with BP using IHC staining. One patient had suboptimal DIF results because of the absence of epidermal tissue in the specimen. Considering that DIF is more sensitive than IHC staining, we reviewed IHC-positive and DIF-negative cases and found that 66.7% of the discordant cases based on IgG detection, 100% of the discordant cases based on C3d detection, and 100% of the discordant cases based on C4d detection were treated as BP cases. Moreover, most cases with negative DIF and positive IHC staining results were considered true BP cases. Fudge et al.^4^ recommended a repeat biopsy after DIF in cases of clinically suspected BP, even if the first DIF result was negative. We suggest that performing IHC staining using FFPE tissue before conducting a second biopsy may be helpful in clinicopathologically suspected BP cases with negative DIF results.

According to the previous report^9^, they observed that C3d has a greater frequency relative to C4d. In our findings, there are more cases which were diagnosed as BP when using DIF C3d than DIF C4d. As is known, C3d and C4d are stable components of complement activation. Though the difference between them is that C4d represents the stable component of the classic complement activation while C3d can be formed either from the classic or alternative complement activation. Bullous pemphigoid is associated complement activation at the dermal–epidermal junction, and Nelson et al.^17^ suggested that complement activation through the classical and alternative pathways have a role in bullous pemphigoid. The reason why C3d and C4d differ in frequency of BP patients is thought to be due to C3d and C4d representing different pathways in complement activation.

However, comparing the result of the DIF and IHC study, IHC C3d had a low sensitivity (41.7%) and high specificity (94.9%) for C3d detection, whereas IHC C4d had a high sensitivity (70.0%) and low specificity (71.4%) to C4d detection. Additionally, IHC C4d had a lower positive predictive value than IHC C3d (24.1% vs. 90.9%). Kassaby et al.^18^ reported a case with negative C3d result and positive C4d result via DIF and IHC studies. They demonstrated that C4d is more stable and persistent in tissue than C3d, as the C4d complement fragment bind covalently to endothelial cells. It may explain our observation that C4d IHC staining could detect C4d more effectively than C3d IHC staining. Also, Wang et al.^19^ explained the reason for false-negative C3d IHC staining may be closely associated with lower anti-BP180 levels that cannot activate complement to detectable levels by IHC. They found that C3d IHC had a sensitivity of only 40% when compared with a positive DIF result, which is consistent with our study.

Based on our results, IHC staining showed good diagnostic value; thus, we hypothesized that a combination of DIF and IHC staining would have a better diagnostic value for BP. However, this combination demonstrated higher sensitivity but lower specificity for detecting deposition than DIF or IHC staining alone. A combination of these two markers could help diagnose false-negative cases more accurately; nevertheless, the results should be carefully interpreted.

IHC staining for diagnosing BP must be performed carefully, and clinicopathological information is vital. It must be conducted on specimens that are histologically consistent with BP in patients clinically suspected to have BP. Moreover, when the diagnostic criteria include linear deposition of two or more markers, the specificity should be 100%, although the sensitivity could be as low as 50%. Therefore, the linear deposition of two or more markers (C3d and IgG, C4d and IgG, C3d and C4d, or C3d, C4d, and IgG) based on IHC staining indicated highly suspected BP.

There are two possible reasons for the discrepancy between DIF and IHC staining results. First, false-negative DIF results caused by technical problems such as difficulties in sample handling and accurate interpretation are possible. Second, the samples used for DIF and IHC staining differed. Generally, DIF is a more sensitive method than IHC staining; therefore, we expected the concordance rate between the two methods to be high. However, when comparing the results of IHC staining and DIF for the detection of a specific marker, we found a fair concordance rate between the two, with ĸ values of 0.227–0.344 and discrepancy rates for IgG, C3d, and C4d of 34.8%, 33.7%, and 28.1%, respectively. Furthermore, most cases with negative DIF and positive IHC staining results were true BP cases. Fudge et al.^4^ reported that the level of autoantibodies present in early lesions was extremely low, perhaps too low for detection, likely resulting in false-negative DIF results. Thus, we speculate that the low amount of antibody in the sample, and not the sensitivity of antibody detection, caused a low concordance rate.

IHC staining offers several advantages. First, additional biopsy was not required. Second, it is a widely used technique; therefore, it is easy to apply in clinical practice. Third, because it is performed on FFPE specimens, histological examination and interpretation of immunopathological results could be performed simultaneously. Detecting linear deposits in areas with histologically suspected BP lesions could lead to a more accurate diagnosis.

This study has some limitations. First, because this was a single-center study, the number of included cases was limited. Second, IHC staining and DIF were not performed on the same samples. An accurate diagnostic value could not be established because of the difference in sample size. With better antigen retrieval techniques becoming available, performing DIF on FFPE skin biopsies may become possible. DIF and IHC staining of the same sample were used to compare the diagnostic value of DIF and IHC staining. Third, we were unable to obtain data on the serum BP antigens.

To the best of our knowledge, our study, which evaluated the diagnostic value of IHC staining as an alternative to DIF for BP diagnosis, includes the second largest number of reported BP cases. Although DIF remains the gold standard, IHC staining may be a useful strategy for BP diagnosis, especially when DIF cannot be performed. Furthermore, we suggest that additional IHC staining of FFPE tissue may be helpful in cases of clinicopathologically suspected BP with negative or suboptimal DIF results, instead of conducting a second biopsy.

## Supplementary Information

Supplementary Tables.

## Data Availability

The datasets analyzed for the study are available from the corresponding author on reasonable request.
