# Supplementary material for: Bullous pemphigoid diagnosis: the role of routine formalin-fixed paraffin-embedded skin tissue immunochemistry
Source: Sci Rep. 2022 Jun 22;12:10519. doi: 10.1038/s41598-022-14950-z (PMC9217790; doi:10.1038/s41598-022-14950-z)
Supplement: Supplementary file 1 — Supplementary Tables. [file 41598_2022_14950_MOESM1_ESM.docx]

**Supplementary Table S1. Comparison of IHC staining and DIF results for IgG**

| **IgG** | **DIF** **(+)** | **DIF** **(-)** | **SUM** |
| --- | --- | --- | --- |
| **IHC (+)** | 20 | 18 | **38** |
| **IHC (-)** | 13 | 36 | **49** |
| **SUM** | **33** | **54** | **87** |

* IHC, immunohistochemical; DIF, direct immunofluorescence

**Supplementary Table S2. Comparison of IHC staining and DIF results for C3d**

| **C3d** | **DIF** **(+)** | **DIF** **(-)** | **SUM** |
| --- | --- | --- | --- |
| **IHC (+)** | 20 | 2 | **22** |
| **IHC (-)** | 28 | 37 | **65** |
| **SUM** | **48** | **39** | **87** |

* IHC, immunohistochemical; DIF, direct immunofluorescence

**Supplementary Table S3. Comparison of IHC staining and DIF results for C4d**

| **C4d** | **DIF** **(+)** | **DIF** **(-)** | **SUM** |
| --- | --- | --- | --- |
| **IHC (+)** | 7 | 22 | **29** |
| **IHC (-)** | 3 | 55 | **58** |
| **SUM** | **10** | **77** | **87** |

* IHC, immunohistochemical; DIF, direct immunofluorescence

**Supplementary Table S4. The result of IHC staining of the other bullous disease**

| **Diagnosis** | **Case No.** | **IgG** | **C3d** | **C4d** |
| --- | --- | --- | --- | --- |
| Linear IgA bullous dermatosis | 1 | Linear | Negative | Negative |
| Dermatitis herpetiformis | 1 | Linear | Negative | Negative |
| Viral infection | 2 | Linear | Negative | Negative |
| Bullous SLE | 1 | Linear | Negative | Negative |
| Stevens-Johnson syndrome | 1 | Linear | Negative | Negative |
